# Different performances in static and dynamic imagery and real locomotion. An exploratory trial

**DOI:** 10.3389/fnhum.2014.00760

**Published:** 2014-10-02

**Authors:** Augusto Fusco, Marco Iosa, Maria Chiara Gallotta, Stefano Paolucci, Carlo Baldari, Laura Guidetti

**Affiliations:** ^1^Department of Movement, Human and Health Sciences, University of Rome Foro ItalicoRome, Italy; ^2^Clinical Laboratory of Experimental Neurorehabilitation, IRCCS Fondazione Santa LuciaRome, Italy

**Keywords:** walking, dynamic motor imagery, human locomotion, healthy, mental representation, chronometry

## Abstract

Motor imagery (MI) is a mental representation of an action without its physical execution. Recently, the simultaneous movement of the body has been added to the mental simulation. This refers to dynamic motor imagery (dMI). This study was aimed at analyzing the temporal features for static and dMI in different locomotor conditions (natural walking, NW, light running, LR, lateral walking, LW, backward walking, BW), and whether these performances were more related to all the given conditions or present only in walking. We have been also evaluated the steps performed in the dMI in comparison with the ones performed by real locomotion. 20 healthy participants (29.3 ± 5.1 years old) were asked to move towards a visualized target located at 10 mt. In dMI, no significant temporal differences respect the actual locomotion were found for all the given tasks (NW: *p* = 0.058, LR: *p* = 0.636, BW: *p* = 0.096; LW: *p* = 0,487). Significant temporal differences between static imagery and actual movements were found for LR (*p* < 0.001) and LW (*p* < 0.001), due to an underestimation of time needed to achieve the target in imagined locomotion. Significant differences in terms of number of steps among tasks were found for LW (*p* < 0.001) and BW (*p* = 0.036), whereas neither in NW (*p* = 0.124) nor LR (*p* = 0.391) between dMI and real locomotion. Our results confirmed that motor imagery is a task-dependent process, with walking being temporally closer than other locomotor conditions. Moreover, the time records of dMI are nearer to the ones of actual locomotion respect than the ones of static motor imagery.

## Introduction

Motor imagery (MI) is defined as a mental rehearsal of an action without its actual performance (Decety, 1996). Evidences have been provided that MI has positive effects on the performance of motor skills, likely by developing of a better implicit understanding about spatial and kinesthetic features required for completing the task (Driskell et al., 1994; Callow et al., 2013). Thereafter, MI has attracted increasing interest of researchers in sport science, psychology, cognitive neuroscience, and, finally, for promoting recovery after a neurological damage in medical sciences worldwide (Müller et al., 2007; Schuster et al., 2011).

Functional imaging studies have shown as motor imagery and motor preparation and execution partially share the same brain networks, as parietal cortex, cortical motor areas, basal ganglia and cerebellum, supporting the hypothesis of functional equivalence (Jeannerod, 1994; Holmes and Collins, 2001; Guillot et al., 2008; Macuga and Frey, 2012).

Many conditions have been pointed out for their role in influencing the MI. For example, MI has been recognized to be closer to real movement when performed with a body posture consistent with the one needed for executing the required action. Conversely, when body posture was not coherent with the imagined movement, the characteristics of similarity were found reduced (Vargas et al., 2004; Fourkas et al., 2006; Saimpont et al., 2012). In addition, the environment and the context in which the MI is performed may improve the mental rehearsal by facilitating the formation of more vivid and precise mental images (Guillot and Collet, 2005; Callow et al., 2006). Recently, it has been hypothesized the existence of an egocentric internal model representing the features of own body and its interaction with the external environment, identified as self-centered mental imagery (Land, 2014). This is a cerebral region deputed to form our conscious percept of a stable world, providing information required by the motor system.

With the introduction of theories related to the Motor Cognition, the same concept of MI is partially changed, revising it as a part of a continuum between preparation and execution of an action where the intentional movement is related to a command following imagined actions (Jeannerod, 2006; MacIntyre and Moran, 2010). Under a pragmatic perspective, the above may explain why MI could impact positively on movements and vice-versa.

Most of the studies on MI have been focused on arm and hand movements. Locomotion received less attention, probably because it involves simulated full-body movements and the concurrent use of environmental information (Kunz et al., 2009). Studies have showed that imagined and physically executed actions share common features. Participants increased the time spent in imagining the walk towards a target located at different distances (Courtine et al., 2004; Plumert et al., 2005; Kunz et al., 2009), consistently with the Fitts’ model on human movement (Fitts, 1954). Further, when participants were asked to walk blindfolded towards a target, they had to combine the motor acts with the imagination (Mittelstaedt and Mittelstaedt, 2001). When MI tasks were assigned adding some constraints, such as the requirement of walking at slower/faster speeds (Bredin et al., 2005) or with shorter/longer steps (Mittelstaedt and Mittelstaedt, 2001) or walking on stilts (Dominici et al., 2009), participants’ motor performances were negatively affected, likely due to the difficulties in imagining an action out of its standard execution.

Recently, the simultaneous coupling of imagination and movement has been introduced in MI trials, referring to it as “dynamic motor imagery” (dMI; MacIntyre and Moran, 2010). This definition concerns about a specific sequence in the MI processes which are associated to movements miming in part those mentally represented, with the same specific features of the action in relation to temporal or spatial invariance (Guillot et al., 2013). The dMI is conceptually different from MI, which is a condition that occurs in the absence of any overt or potential movement (Guillot and Collet, 2010). However, despite its theoretical definition, trials have evidenced that, during MI, a subliminal muscular activity was possible, suggesting by it that the motor control is not completely inhibited (Guillot et al., 2007; Lebon et al., 2008). Consequently, it has been suggested that a motor output is possible and may be included within MI (Morris et al., 2005).

In some preliminary studies where imagery and movement were associated, Callow et al. (2006) reported as high-level junior skiers who moved the body from side to side, simulating the actions during a skiing competition, experienced more vivid images and increased their confidence in the performance of athletic movements. Vergeer and Roberts (2006) reported the improvement of stretching exercises in terms of flexibility in participants with more vivid imagery of their exercises. Finally, a recent study has shown that the technical performance can be improved in active high jumpers (Guillot et al., 2013). These studies have led some authors to conclude that applying a dynamic support to the imagination could result in improvements of the performance (Smith et al., 2007). Nevertheless, at the current stage data are still poor.

The first aim of this study was to evaluate if dynamic MI is equivalent or superior to MI in representing temporal features of real execution for different types of locomotion. These different locomotor conditions were normal walking, light running, lateral walking and backward walking. Because MI performances have shown to be related to the usual physical practice (Aglioti et al., 2008), we have also hypothesized that both MI and dMI could be closer to real performances during normal walking than into the other tested locomotor conditions.

## Materials and methods

### Participants and protocol

Twenty healthy volunteers were enrolled in this study (8 males; 12 females; mean age: 29.3 ± 5.1 years). They were asked to stand on a line marked on the floor in front of another line taped on the ground, at a distance of at 10 mt, unknown by the participants.

They were asked to imagine achieving the target in one of four possible randomized conditions: normal walking (W), light running (LR), lateral walking (LW), and backward walking (BW). Imagery could be accompanied by stepping in place (dynamic motor imagery, dMI) or not (static motor imagery, sMI) the required type of locomotion.

After having performed these tasks for all requested conditions, individuals were asked to really perform the task, going towards the target according to the randomizations sequence. The randomized sequence of locomotor types was repeated for each one of the three tasks, sMI, dMI, real performance (RP), performed in this order.

To avoid some possible learning and/or cognitive influencing effects, we tested naïve subjects to the requested tasks. No verbal information were given to the participants about the target distance and their performances, as similarly conducted in previous studies (Bredin et al., 2005; Iosa et al., 2012a). The experimental trial was conducted in the same indoor environment, to avoid possible influence of different settings (Lappin et al., 2006; Iosa et al., 2012a).

The study was conducted in accordance with the Declaration of Helsinki about experiments on human subjects. This study was approved by the local ethical committee of our institution (Research Rehabilitation Hospital) where all tests were carried out. Signed informed consent was obtained from each participant.

### Measurement settings

The main measures were related to temporal features among static imagery, dynamic imagery and actual performance for all the analyzed forms of locomotion. The response time data was the main outcome measure for all behavioral experiments. Consistently with previous studies, we compared the duration of the performance between real and imagined motor acts (Collet et al., 2011). In the assessment of MI, the chronometric tests have widely proved to be a reliable technique both in healthy subjects and in patients (Malouin et al., 2008). Execution time during MI is close to that of actual execution (Guillot and Collet, 2005). We do not use self-report inventories to measure MI performances in order to avoid the risk to involve subject’s conscious awareness, potentially altering the data (Collet et al., 2011).

During sMI, time was measured using a chronometer by a professional sports chronograph digital timer stopwatch (JUNSD^®^). Participants were instructed to use the chronograph by themselves to mark the beginning and the end of each trial, as already performed in previous study (Lebon et al., 2012). During dMI, measures were taken with accelerometers. They work by means of a wearable inertial sensor device (FreeSense, Sensorize s.r.l., Rome; sampling frequency = 100 Hz), located inside an elastic belt on an area of their back corresponding to the L2-L3 spinous processes, close to the body center of mass.

Accelerometric inertial sensor, a suitable simple quantitative technique, was used to objectively assess the dynamic gait stability of subjects during walking and to estimate spatiotemporal parameters in many previous studies both in healthy subjects and in patient with several diseases (Iosa et al., 2012b,c,d, 2013). This device is lightweight (93 g) and contains a triaxial accelerometer to measure accelerations along the three body axes (antero-posterior, AP; latero-lateral, LL; and cranio-caudal, CC) and three gyroscopes to measure angular velocities around the above axes. We used it to measure movement time and for estimating the number of performed steps (corresponding to the negative peak of antero-posterior acceleration) in place or along the pathway in dMI and RP. The acceleration data were recorded during consecutive steps and these signals were analyzed after the subtraction of their mean values and after low-pass filtering at 20 Hz and were summarized in parameters for each body axis, as commonly used in previous studies (Iosa et al., 2012d,e).

### Statistical analysis

Means and standard deviations were computed for all the investigated parameters (demographic data of subjects, spatio-temporal results obtained by tests). Repeated measure analysis of variance using as within subject factor the task (sMI, dMI, RP) were performed using time as dependent variable and number of steps performed by subjects for each one of the four locomotion condition (normal walking, light running, backward walking and lateral walking). *Post-hoc* analyses were performed when needed, correcting the level of significance in accordance to Bonferroni correction (*p* < 0.025); for all the other analyses this level was set at 0.05. Pearson’s correlation coefficient (*R*) was computed for evaluating the relationship between the mean values computed into the four different locomotor types between sMI and RP, dMI and RP, and between sMI and RP.

## Results

**Figure 1:**
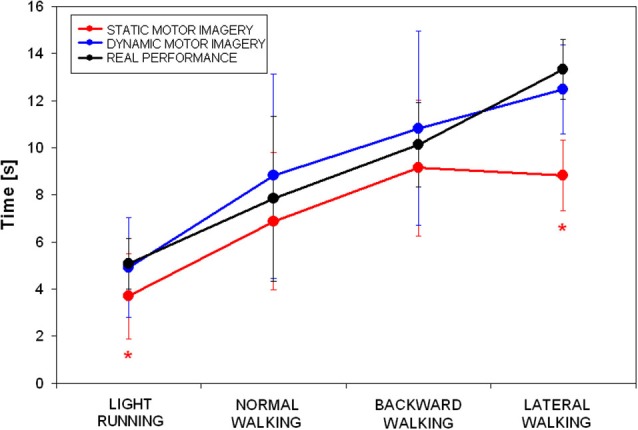
Mean and standard deviations of time spent during the three tasks (static motor imagery in red, dynamic motor imagery in blue and actual locomotion in black) in the four locomotor conditions (* *p* < 0.025 in the *post hoc* showed in Table 1).

The mean time spent by participants during the three tasks (sMI, dMI, RP) in the four locomotor conditions (light running, normal walking, backward walking and lateral walking) is shown in Figure 1.

**Table 1:** Results of repeated measure analysis of variance and relevant *post-hoc* Analyses on time spent by subjects in the three tasks: static motor imagery (sMI), dynamic motor imagery (dMI) and real performance (RP) (* *p* < 0.025).

Time	Analysis of variance		*Post-hoc*		
**Locomotor task**	***F***	***P***	**sMI vs. RP**	**dMI vs. RP**	**sMI vs. dMI**
**Light running**	16.011	**<0.001**	<0.001*	0.636	<0.001*
**Normal walking**	9.433	**<0.001**	0.041	0.058	<0.001*
**Backward walking**	**2.494**	0.096	—	—	—
**Lateral walking**	11.392	**<0.001**	<0.001*	0.487	<0.001*

Repeated measure analysis of variance showed that significant differences were found for all conditions among tasks, except for backward walking (see Table 1). *Post-hoc* analyses revealed that: (i) time was not different between dMI and RP for any of the locomotor condition; (ii) time was different between sMI and RP for light running and lateral walking; and (iii) time was different between sMI and dMI for light running, normal and lateral walking.

The temporal correlation between simulated dMI and RP resulted statistically significant (*R* = 0.972, *p* = 0.028, as evident in Figure 1). The correlation between sMI and RP was lower and not statistically significant (*R* = 0.890, *p* = 0.110). As shown in Figure 1, it was mainly due to an underestimation of time needed to achieve the target by lateral walking during static motor imagery.

**Figure 2:**
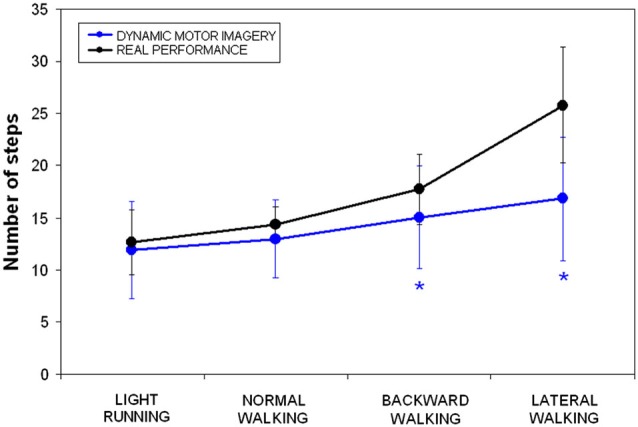
Mean and standard deviations of number of steps during dynamic imagery (blue) and actual (black) locomotion in the four conditions (* *p* < 0.025 in the *post-hoc* analyses).

Number of steps could be analyzed only in dMI and RP. Repeated measure analysis of variance showed significant differences in lateral walking (*F* = 22.733, *p* < 0.001) and in backward walking (*F* = 5.087, *p* = 0.036), whereas neither in normal walking (*F* = 2.609, *p* = 0.124) nor in running (*F* = 0.770, *p* = 0.391) significant differences were observed in terms of performed steps between dMI and real performances. As clearly shown in Figure 2, there was a significant correlation in terms of number of steps between dMI and RP (*R* = 0.991, *p* = 0.009).

## Discussion

The first aim of this study was to investigate whether dMI was superior to static motor imagery into imagining the achievement of a target placed at given distance (10 mt) in different locomotor conditions. Our results clearly supported this hypothesis, with similar temporal performances between dMI and real performances. Conversely, the time spent during sMI, resulted significantly underestimated with respect to the one really needed in light running and lateral walking. Individuals showed a good capacity to mentally estimate the needed time only for normal and backward walking.

We had hypothesized that normal walking could be more easily performed than other types of locomotion, according to idea that usual physical practice could enhance MI (Aglioti et al., 2008), probably for a greater neural overlap (Guillot et al., 2013). This hypothesis was supported by our results. Surprisingly, a temporal similarity between sMI and RP was obtained also during backward walking. Despite some differences, the kinematics of forward and backward human locomotion are quite similar (Viviani et al., 2011), and it could explain our results.

For the other locomotor conditions (light running and lateral walking), significant differences were found between sMI and RP. This result suggests that motor imagery could be a process dependent on the motor act to imagine. At the same time, this dependency was not significant when imagery is coupled with external movements miming in part those mentally represented.

For dMI, we have also analyzed the number of simulated steps. They resulted significantly different from those really performed to achieve the target only during backward and lateral walking. Especially, for this last condition, the number of steps was roughly halved in respect of actual one. Hence, lateral walking resulted the task more difficult to be imagined. Differently from the other locomotor conditions, the movement of one leg never pass over the other leg position during actual lateral walking. Likely, participants did not take into account this peculiarity when they had been asked to imagine to perform this task.

Our results confirm that the temporal features of dMI are similar to those of RP even for complex movements (Olsson et al., 2008). At the same time, this was also applicable for spatial features (performed steps) during normal walking and light running. The result could be due to the fact that dMI, more than sMI, involves brain networks with a more vivid imagination of movement with a spatial updating and re-calibration of the distance perception, that are critical factors in locomotor performance, especially for less complex movements (or, in our case, for more usual movements) (Guillot et al., 2013).

Our results could be also explained on the basis of the theory of internal models. Actually, with regards to the latter, it has been hypothesized the existence of a specific internal model related to walking, called locomotor body schema (Dominici et al., 2009). Spino-cerebellar neuronal networks could encode information of limb length, combining them with information of limb kinematics, for computing a predictive measure of step length and hence of walked distance (Ivanenko et al., 2011).

During MI, the body schema could provide the needed information about limb length, independently if in static or dynamic condition, however, MI could benefit of simulation of limb kinematics, for the proprioceptive and sensory inputs, together with possible information related to the efferent motor commands. Hence, the processing of this information may differ significantly in static and dynamic imagery due to a diversity of the available information, resulting in a better temporal (and for usual locomotion also spatial) correlation between dMI and RP. This locomotor body schema should be taken into account also perceived distance and spatial updating, in accordance with the walking imagery (Loomis et al., 1992; Kunz et al., 2009). However, it should be noted that Guillot et al. have reported that dMI was effective also for upper limb movements: mimicking the gestures of the upper limbs, the athletes were able to improve their kinesthetic ability (Guillot et al., 2013). Our results on dMI support this idea and confirm the functional equivalence between action and imagination (Jeannerod, 1994; Decety, 1996).

Dynamic motor imagery showed temporal similarity with real performances independently by the locomotor conditions, but from a spatial point of view this similarity was limited to walking and running. In consideration of our results, this locomotor body schema seemed to be motor act-dependent and more effective for these two locomotor acts. There are two possible explanations for these results: backward and lateral walking are uncommon in normal daily-living and/or the direction of body progression did not coincide with the front of the body. Lateral and backward walking are more common during such type of sports (for example, basketball, soccer, volley) and future studies could investigate if athletes have better performances than non-athletes in these other types of locomotion. Globally, MI has been found to be more effective in athletes (Rushall and Lippman, 1998; Bredin et al., 2005; Callow et al., 2013).

Limitations of this study were probably due to some uncontrolled conditions. For example, lateral walking was explained to the participants as a lateral movement without leg crossing. Despite of it, and despite of the fact that they correctly performed the test as asked during RP, they be-halved the number of steps into the imagery tasks, such as they imagined both the legs as doing propulsive forward movements. Then, we did not administered a questionnaire about the vividness of motor imagery, as in previous study (Guillot et al., 2013). Therefore, future studies should focus on the possible differences between simple and complex movements, both for healthy and patients, analyzing the different motor impairments, also using appropriate measures (Beauchet et al., 2010). Differently from previous researches, our study has the worth to have investigated the MI in target-directed different locomotor conditions, possible in the real-life situations and important in such sports.

In conclusion, our study approach was innovative, focusing on the concepts of motor imagery in locomotion. Our results confirmed that motor imagery is a task-dependent process also for the human locomotion. In fact, imagined and executed walking are more temporally closer respect than other locomotor conditions, showing a functional equivalence. Moreover, the time records of dMI are nearer to the ones of actual locomotion respect than the ones of static motor imagery, revealing a potential important role of dMI for improving performances.

## Conflict of interest statement

The authors declare that the research was conducted in the absence of any commercial or financial relationships that could be construed as a potential conflict of interest.
